# Superior RdRp Function Drives the Dominance of Prevalent GI.3 Norovirus Lineages

**DOI:** 10.3390/microorganisms14010011

**Published:** 2025-12-19

**Authors:** Qianxin Lu, Huisha Du, Xin Jiang, Bingwen Zeng, Tianhui Li, Ying-Chun Dai

**Affiliations:** Guangdong Provincial Key Laboratory of Tropical Disease Research, Department of Epidemiology, School of Public Health, Southern Medical University, Guangzhou 510515, China; 3180090131@smu.edu.cn (Q.L.); 3170090013@i.smu.edu.cn (H.D.); jx22320103@smu.edu.cn (X.J.); 3190090022@smu.edu.cn (B.Z.); lth22420095@smu.edu.cn (T.L.)

**Keywords:** GI.3 norovirus, viral evolution, RNA-dependent RNA polymerase, enzyme kinetics, Bayesian phylogenetic analysis

## Abstract

The GI.3 norovirus is the most detected and recombinant-rich genotype within genogroup I, yet the mechanistic basis for its epidemiological success remains poorly understood. This study integrates Bayesian evolutionary analysis with in vitro enzymology to investigate the link between RdRp function and the evolutionary dynamics of GI.3 NoV. We analyzed 831 GI.3 sequences, finding that prevalent strains (GI.3[P3] and GI.3[P13]) exhibited significantly higher evolutionary rates in both the RdRp and VP1 genes than non-prevalent strains (GI.3[P10] and GI.3[P14]). While the RdRp gene displayed a strong molecular clock signal, the VP1 gene’s evolution was more complex, showing cluster-specific trends. Functionally, the RdRps from prevalent strains demonstrated superior enzymatic activity and substrate affinity (K_m_: GI.3[P13] = 0.092 mM; GI.3[P3] = 0.176 mM) compared to non-prevalent strains (K_m_: GI.3[P14] = 0.273 mM). Notably, GI.3 RdRp required higher manganese ion concentrations for optimal activity than previously reported for GII strains, suggesting a potential biochemical constraint. Our findings demonstrate a clear correlation between RdRp enzymatic efficiency, evolutionary rate, and strain prevalence. We propose that a highly active RdRp may potentially accelerate VP1 evolution and confer a replicative advantage, underpinning the dominance of specific GI.3 lineages. This work provides crucial experimental evidence linking viral polymerase function to evolutionary and epidemiological outcomes.

## 1. Introduction

Norovirus (NoV) persists as a pathogen of substantial global public health significance, accounting for over 21 million episodes of acute gastroenteritis (AGE) and more than 200,000 deaths annually [1]. A critical challenge in controlling NoV is its rapid evolution, with novel variants capable of triggering global epidemics emerging every 2–3 years, thereby posing significant challenges to vaccine design and development [2]. NoV is a single-stranded positive-sense RNA virus. Its genome contains three open reading frames (ORFs): ORF1 encodes non-structural proteins, including the RNA-dependent RNA polymerase (RdRp), while ORF2 and ORF3 encode the major and minor capsid proteins (VP1 and VP2), respectively [3,4].

Frequent genetic recombination is a core driving force behind NoV diversity and global prevalence [5,6]. Notably, recombination occurs primarily at a hotspot within the ORF1/ORF2 overlap, facilitating the exchange of RdRp and VP1 genes among strains [7]. This generates viral lineages with novel genotype combinations, making the coordinated characterization of RdRp and VP1 essential for understanding viral emergence and spread. The RdRp, as the central enzyme for viral RNA synthesis, is a key determinant of replication fidelity and evolutionary rate. Multiple studies have demonstrated that epidemic strains of NoV possess higher RdRp evolutionary rates and replication activities compared to non-epidemic strains, underscoring a strong correlation between RdRp function and epidemic potential [5,7,8]. Conversely, the VP1 capsid dictates host tropism and immune evasion by interacting with histo-blood group antigens (HBGAs), and amino acid mutations in its P2 subdomain can alter receptor binding and facilitate escape from population immunity [9,10,11].

Although GII genogroup NoVs are the predominant cause of global outbreaks [12], the public health threat posed by GI NoVs is likely underestimated. Infections with GI NoVs are highly prevalent, as evidenced by high seropositivity rates (35.0–38.8%) of blockade antibodies in our previous study [13] and their frequent detection in environmental and food surveillance [14,15,16,17,18]. This is particularly true in regions with intensive shellfish production and consumption, where monitoring is heightened. Among GI NoVs, the GI.3 genotype is of particular interest: it is the most frequently detected (58%) and exhibits the greatest diversity of recombinant strains [19]. The two most common lineages, GI.3[P3] and GI.3[P13], engage in frequent recombination [20]. However, the molecular mechanisms that underpin the epidemiological success of these specific GI.3 lineages remain elusive. It is unknown whether their prevalence is linked to superior replicative fitness conferred by their RdRp, as has been observed in prevalent GII strains. This study tests the hypothesis that the prevalence of specific GI.3 NoV strains is driven by enhanced RdRp enzymatic activity, which in turn accelerates viral evolution. We integrate large-scale phylogenetic and evolutionary rate analyses with detailed in vitro biochemical characterization of RdRp function across different RdRp genotypes of GI.3 NoV. Our objective is to establish a direct link between the replication machinery’s efficiency, the pace of genome evolution, and epidemic outcomes, thereby providing a mechanistic framework for understanding NoV strain emergence and a foundation for future antiviral strategies.

## 2. Materials and Methods

### 2.1. Sequence Data Collection

All nucleotide sequences of GI.3 NoV were retrieved from the National Center for Biotechnology Information (NCBI) GenBank database (http://www.ncbi.nlm.nih.gov/genbank, accessed on 27 March 2025). To ensure data quality, the initial dataset was rigorously curated by excluding food and environmental samples. Such samples are prone to contamination with non-human viruses (e.g., phages) and to high background diversity, which can complicate analyses and lead to misleading conclusions when tracing human outbreaks. This step enhances the reliability of downstream evolutionary analyses restricted to human strains. The remaining sequences were genotyped using the NoV Typing Tool (Ver 2.0), and only those unambiguously classified as GI.3 were retained. Sequences with an excessively high proportion of ambiguous bases (>2%), or >99% pairwise identity (potential duplicates) were removed. Finally, complete or near-complete sequences for the VP1 and RdRp regions of GI.3 NoV were selected to construct separate datasets for evolutionary analysis. Associated metadata, including detection date and geographic origin, were compiled. Detailed information on the final sequences used was provided in Table S1.

### 2.2. Phylogenetic and Evolutionary Rate Analysis

Multiple sequence alignments of the VP1 and RdRp datasets were performed using MEGA 11.0 (Biodesign Institute, Arizona State University, Tempe, AZ, USA) [21]. The best-fit nucleotide substitution model for each dataset was determined using the Bayesian Information Criterion (BIC) within MEGA (models detailed in Table S2). Maximum likelihood (ML) phylogenetic trees were constructed separately for each region with 1000 bootstrap replicates. The resulting trees were visualized using the online platform Chiplot [22].

Evolutionary rates based on time-scaled phylogenies were estimated using a Bayesian Markov Chain Monte Carlo (MCMC) approach implemented in BEAST v1.10.4 (Institute of Evolutionary Biology, University of Edinburgh, Edinburgh, UK; Centre for Computational Evolution, University of Auckland, Auckland, New Zealand) [23,24]. The same nucleotide substitution models as in the ML analysis were applied. To select the most appropriate molecular clock and demographic models, we compared the combination of two clock models (strict and uncorrelated relaxed lognormal clock) and three tree priors (constant population, exponential growth, and Bayesian skyline) using path sampling/stepping-stone sampling. The best-fitting model for each dataset, based on marginal likelihood estimation, is detailed in Table S3. For each analysis, the MCMC chain was run for 400 million generations, sampling every 40,000 steps. Convergence was assessed using Tracer v1.5.3 (Institute of Evolutionary Biology, University of Edinburgh, Edinburgh, UK; Centre for Computational Evolution, University of Auckland, Auckland, New Zealand), ensuring all parameters reached effective sample sizes (ESS) > 200 after a 10% burn-in was discarded.

To evaluate the strength of the temporal signal for subsequent molecular clock analysis, a root-to-tip regression analysis was conducted. Based on the ML phylogenetic trees, inter-tip divergence distances were calculated in TempEst v1.5 (Institute of Evolutionary Biology, University of Edinburgh, Edinburgh, UK; Centre for Computational Evolution, University of Auckland, Auckland, New Zealand) and regressed against the collection dates of the strains to assess clock-like evolution for the VP1 and RdRp genes.

### 2.3. RdRp Protein Expression and Purification

One representative strain for each of the four major RdRp genotypes (GI.3[P3], GI.3[P10], GI.3[P13], and GI.3[P14]) was selected for functional characterization (GenBank accession numbers: PP594195.1, MT008457.1, MH218730.1, and MW445537.1). The RdRp coding sequence for each was synthesized with an N-terminal 6 × His tag and cloned into the pET28a vector (Beijing Genomics Institute, Beijing, China). The recombinant plasmids were transformed into *E. coli BL21 (DE3)* for protein expression. The transformed bacterial culture was first grown overnight (37 °C, 220 rpm, 16 h), then subcultured at a 1:1000 dilution into fresh LB broth (Solarbio, Beijing, China) and incubated at 37 °C for 2–3 h. Expression was induced with 0.5 mM isopropyl β-D-1-thiogalactopyranoside (IPTG, Genview, Tallahassee, FL, USA) when the OD_600_ (optical density at 600 nm) reached 0.4–0.6, followed by incubation at 16 °C for 16 h). Cells were harvested, lysed by ultrasonication, and the soluble fraction was collected by centrifugation. Following expression, the soluble fraction was first purified by Ni-affinity chromatography (BBI, Cardiff, UK) with imidazole-gradient elution. The eluate was then buffer-exchanged and concentrated using ultrafiltration, followed by anion-exchange chromatography (Solarbio, Beijing, China) to remove residual nucleic acids [25]. This process yielded RdRp protein of high purity and concentration, which was aliquoted and stored at −80 °C.

### 2.4. RdRp Enzymatic Activity Assay

RdRp activity was measured using a de novo RNA synthesis assay, which quantifies the production of double-stranded RNA (dsRNA) [26]. The standard reaction mixture (25 μL) contained 40 μg/mL poly(C) RNA template (Aladdin, Shanghai, China), 0.5 mM GTP (Beyotime, Beijing, China), 1 mM MnCl_2_ (Coolaber, Beijing, China), 5 mM dithiothreitol (DTT, Leagene, Beijing, China), 20 mM Tris-HCl (pH 7.5,Leagene, Beijing, China), and a specified amount of purified RdRp [8]. To establish optimal reaction conditions, we empirically determined the effects of incubation time (0–120 min), enzyme concentration (0–5 μg), MnCl_2_ concentration (0–8 mM), and temperature (5–60 °C). After the reaction, the mixture was transferred to a black 96-well plate, mixed with PicoGreen^®^ dsDNA Quantitation Reagent (YEASEN, Shanghai, China), a fluorescent dye specific for dsRNA, and fluorescence intensity was measured (excitation/emission: 485/520 nm). The relative fluorescence unit (RFU) was calculated by subtracting the mean fluorescence of a negative control (no GTP) from each sample value.

### 2.5. Enzyme Kinetics Assay

To determine the Michaelis-Menten kinetic parameters, the standard reaction was performed with varying GTP concentrations (0–0.6 mM) while keeping other conditions constant (5 μg protein, 1 mM MnCl_2_, 30 °C, 20 min). The initial reaction velocities (V_0_) derived from RFU values were plotted against substrate concentrations. The Michaelis constant (K_m_) and the maximum velocity (V_max_) for each RdRp variant were obtained by fitting the data to the Michaelis-Menten equation using non-linear regression in GraphPad Prism v10.0 (GraphPad Software, Boston, MA, USA). The goodness of fit (R^2^) for all analyses was set as >0.9.

## 3. Results

### 3.1. Genomic Epidemiology of GI.3 NoV

**Figure 1:**
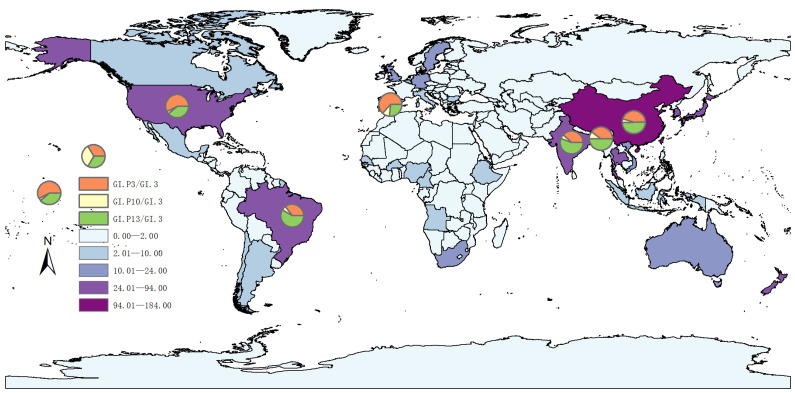
Global distribution of GI.3 norovirus sequences.

A total of 831 GI.3 NoV sequences (1969–2024) were retrieved in this study, of which 431 had both VP1 and RdRp genotyping information. The prevalent strains were GI.3[P3] (52.43%, 226/431) and GI.3[P13] (41.76%, 180/431), while GI.3[P10] (4.18%) and GI.3[P14] (0.93%) were rare (Table 1). Geographically, most sequences originated from Asia (*n* = 486) and Europe (*n* = 121). In Asia, GI.3[P13] (47.79%) was dominant, whereas GI.3[P3] (68.85%) was predominated in Europe (Figure 1, Table S4).

### 3.2. Genetic Characterization

**Figure 2:**
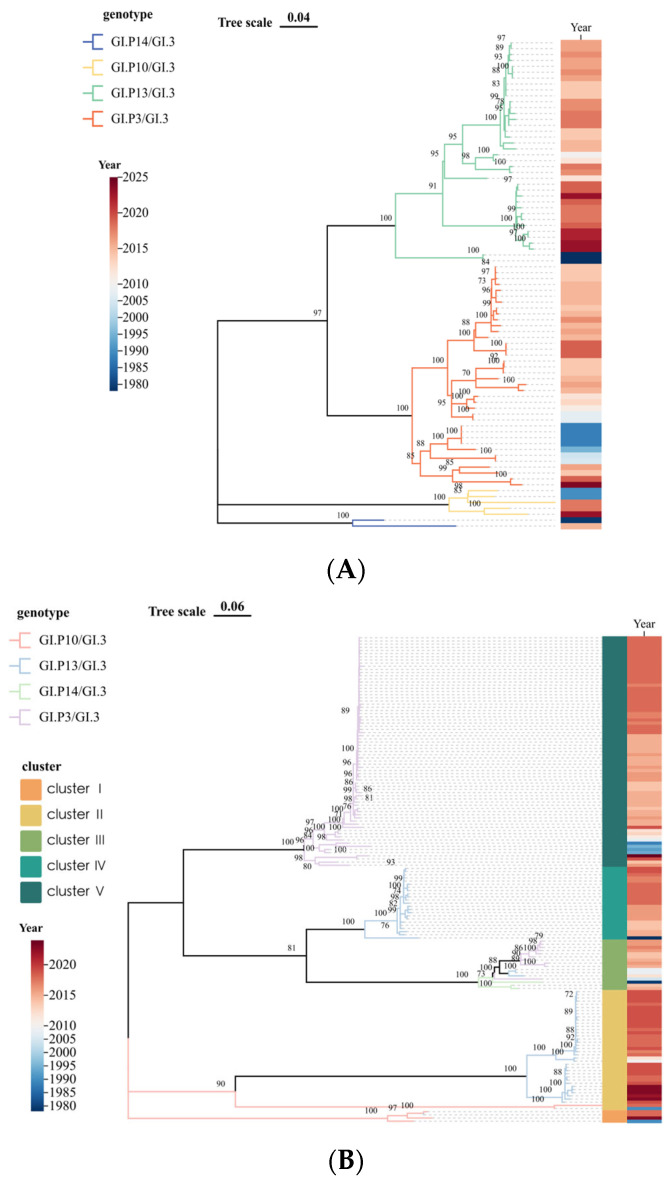
Maximum Likelihood phylogenies of GI.3 norovirus. (**A**) RdRp gene nucleotide sequences. (**B**) VP1 gene nucleotide sequences. Branch colors indicate RdRp genotypes. Heatmap represents collection years.

Maximum likelihood phylogenetic trees were constructed using 154 (VP1) and 83 (RdRp) complete or nearly complete sequences. Sequences containing excessive ambiguous bases or with >99% pairwise identity were excluded prior to analysis. The RdRp nucleotide tree showed distinct clustering by P-genotype, with GI.3[P13] exhibiting a closer evolutionary relationship to GI.3[P3] than to GI.3[P10] or GI.3[P14] (Figure 2A). In contrast, the VP1 tree revealed five clusters with intermingled RdRp genotypes: Cluster I (GI.3[P10]), Cluster II (GI.3[P13] and some GI.3[P10]), Cluster III (GI.3[P3], some GI.3[P13], and all GI.3[P14]), and Clusters IV and V (GI.3[P13] and GI.3[P3], respectively) (Figure 2B).

### 3.3. Time-Scale Evolutionary Characterization

The overall evolutionary rates of the GI.3 NoV RdRp and VP1 genes were comparable (Table 2). Notably, the evolutionary rates of the RdRp genes were significantly higher than those of their VP1 counterparts within each genotype. However, genotype-specific analysis revealed that the prevalent GI.3[P3] strain had the highest evolutionary rates for both RdRp (5.26 × 10^−3^ subs/site/year) and VP1 (3.76 × 10^−3^), while the non-prevalent GI.3[P10] showed the lowest rates.

**Figure 3:**
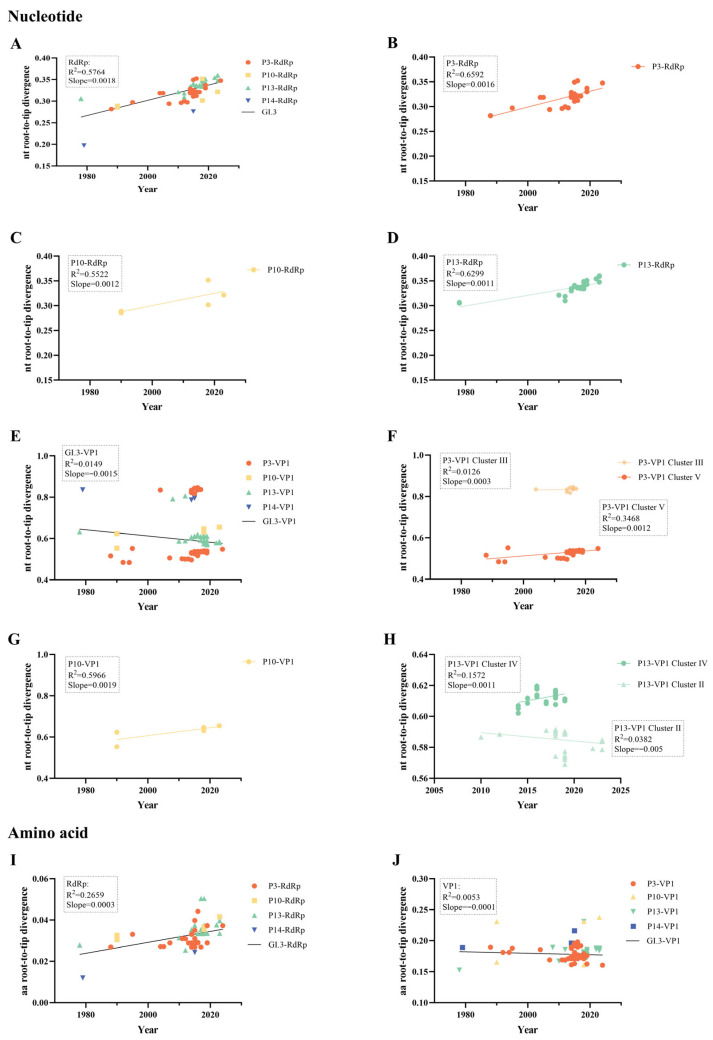
Temporal signal analysis of GI.3 norovirus evolution. (**A**–**D**) Nucleotide root-to-tip divergence of RdRp gene. (**E**–**H**) Nucleotide root-to-tip divergence of VP1 gene. (**I**,**J**) Amino acid root-to-tip divergence. Linear regression lines shown in black. “P3”, “P10”, “P13”, and “P14” refer to genotypes GI.3[P3], GI.3[P10], GI.3[P13], and GI.3[P14], respectively.

Nucleotide root-to-tip regression showed a moderately strong temporal signal for the RdRp gene (R^2^ = 0.5764) (Figure 3A–D). In contrast, the VP1 gene exhibited a complex pattern: while GI.3[P3] VP1 (Cluster V) and GI.3[P13] VP1 (Cluster IV) showed positive mutation trends, GI.3[P13] VP1 (Cluster II) displayed a negative trend (Figure 3E–H). Amino acid root-to-tip plots showed negligible mutation accumulation over time for both genes (Figure 3I,J).

### 3.4. Expression and Verification of GI.3 NoV RdRp Proteins

All four recombinant His-tagged RdRp proteins were successfully expressed in *E. coli BL21* and purified to homogeneity using a two-step Ni-NTA and Q Sepharose chromatography protocol, yielding soluble proteins of the expected ~58 kDa size (Figures S1 and S2).

### 3.5. Characterization of GI.3 NoV RdRp Activity

**Figure 4:**
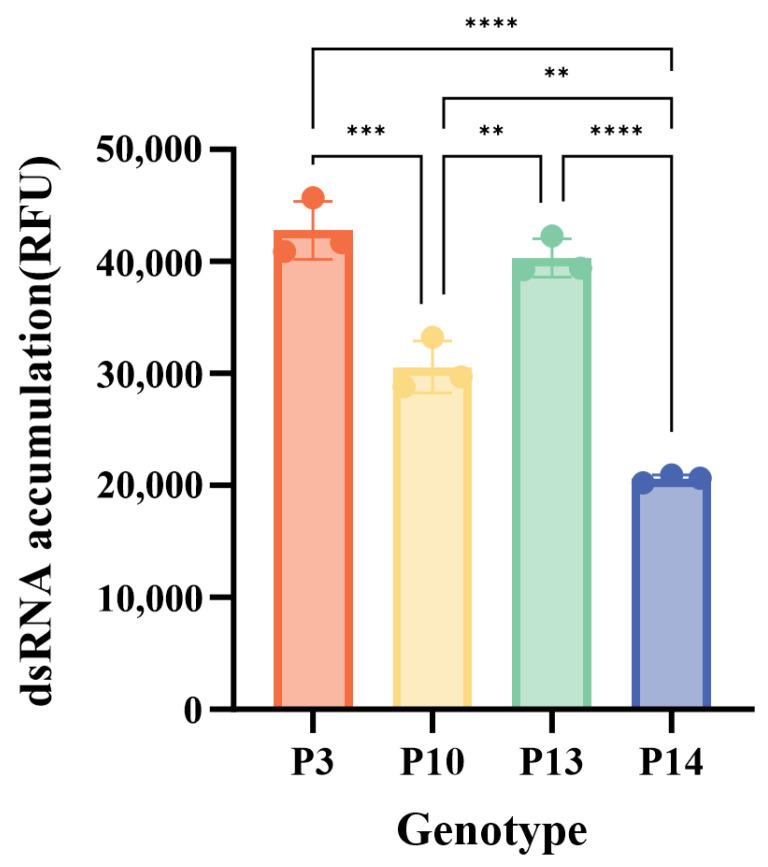
Enzymatic characterization of GI.3 norovirus RdRp variants. dsRNA synthesis was measured under varying standard conditions for genotype comparison. “P3”, “P10”, “P13”, and “P14” refer to genotypes GI.3[P3], GI.3[P10], GI.3[P13], and GI.3[P14], respectively. ** *p* < 0.01, *** *p* < 0.001, **** *p* < 0.0001.

The catalytic activity of the four purified GI.3 NoV RdRp proteins was validated using a poly(C)-dependent de novo RNA synthesis assay. Under standardized reaction conditions, the fluorescence signals increased over time, confirming dsRNA synthesis (Figure S3A). The relative fluorescence unit (RFU) growth rates stabilized within the first 20 min; therefore, a 20 min reaction time was adopted. Optimization experiments determined the ideal conditions: 5 μg protein (Figure S3B), 1 mM MnCl_2_ (Figure S3C), and a reaction temperature of 30 °C (Figure S3D). Under these established conditions (40 μg/mL poly(C), 0.5 mM GTP, 1 mM MnCl_2_, 5 mM DTT, 20 mM Tris-HCl (pH 7.5), 5 μg RdRp, 30 °C for 20 min), the enzymatic reaction rates of the prevalent strains GI.3[P3] and GI.3[P13] were significantly higher than those of the non-prevalent strains GI.3[P10] and GI.3[P14] (*p* < 0.01) (Figure 4).

### 3.6. Substrate Kinetics of GI.3 NoV RdRp

**Figure 5:**
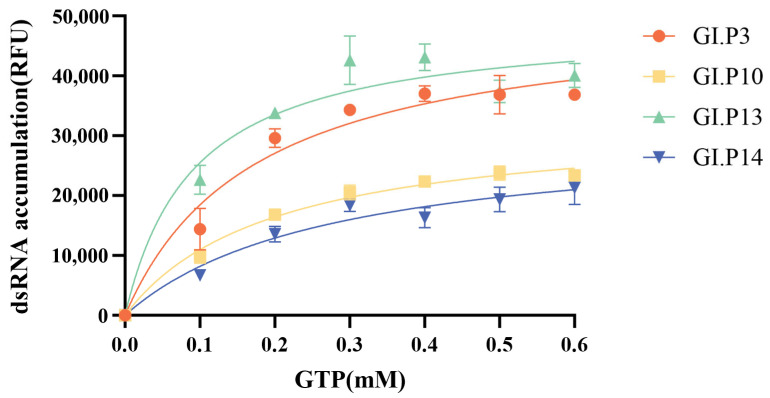
Steady-state kinetics of GI.3 norovirus RdRp variants. Michaelis-Menten plots show initial velocity versus GTP concentration. Error bars represent standard deviation from triplicate measurements. “P3”, “P10”, “P13”, and “P14” refer to genotypes GI.3[P3], GI.3[P10], GI.3[P13], and GI.3[P14], respectively.

Substrate kinetics were analyzed by measuring RdRp activity across GTP concentrations (0–0.6 mM). The Michaelis constants (K_m_) for the four RdRp variants ranged from 0.092 to 0.273 mM, with all data fitting well to the Michaelis-Menten equation (R^2^ > 0.9) (Figure 5, Table 3). The prevalent strain GI.3[P13] showed the highest substrate affinity (lowest K_m_ = 0.092 mM), followed by GI.3[P3] (K_m_ = 0.176 mM), while the non-prevalent GI.3[P14] exhibited the lowest affinity (highest K_m_ = 0.273 mM). Furthermore, GI.3[P3] achieved the highest V_max_ value (50,817 RFU), 1.7-fold greater than that of GI.3[P14]. According to enzymatic kinetic principles, these results indicate that the prevalent strains possess not only superior substrate affinity but also enhanced catalytic efficiency.

## 4. Discussion

Recent surveillance reports indicate an increase in GI.3-associated outbreaks [27,28,29,30]. Our sequence collection reflects this, with GI.3[P3] and GI.3[P13] dominating the dataset (Table 1) consistent with the genotype distribution reported by Barclay L [31]. These sequences originated primarily from Asia and Europe, with distinct regional distributions: GI.3[P13] co-circulates with GI.3[P3] in Asia, while GI.3[P3] predominates in Europe (Figure 1, Table S4). This geographic disparity may be driven by differences in local population susceptibility and climate [32], or may simply reflect a surveillance bias.

Phylogenetic analysis revealed distinct evolutionary patterns for the RdRp and VP1 genes. While the RdRp tree showed clear segregation by P-genotype, the VP1 tree exhibited intermingled clustering, a pattern consistent with the occurrence of historical recombination events. Particularly notable was the closer evolutionary relationship between the GI.3[P3] and GI.3[P13] polymerases compared to other genotypes. This phylogenetic proximity likely facilitates template-switching events between these lineages, supporting previous surveillance data indicating that GI.3[P3] and GI.3[P13] are dominant and engage in frequent recombination [33]. Furthermore, the formation of geographically distinct VP1 clusters, such as for GI.3[P13] strains in Asia versus Europe/America, suggests that following recombination, viral subpopulations evolve independently under different local pressures, forming transmission networks with regional characteristics [34,35].

A key finding is the tiered evolutionary rate among GI.3 genotypes (GI.3[P3] > GI.3[P13] > GI.3[P10]), which correlates directly with their epidemiological prevalence. This pattern suggests the viral replication machinery significantly contributes to capsid protein diversification, aligning with observations in GII NoVs where novel polymerases can accelerate VP1 evolution [36]. Our data corroborate that GI.3 is the fastest-evolving genotype within genogroup I [37,38]. We conducted stratified analyses of the GI.3[P3] and GI.3[P13] strains by their primary sampling regions and years (Table S5). Across both Asia and Europe, GI.3[P3] consistently showed higher evolutionary rates than GI.3[P13]. This difference was particularly pronounced during 2011–2015. These findings reinforce the notion that divergence in the RdRp gene may influence VP1 evolution. However, from 2016–2020, the evolutionary rate of GI.3[P13]-VP1 increased to match that of GI.3[P3]. This shift coincides temporally with documented outbreaks of GI.3[P13] in localized settings [20,32], often during colder seasons. The ability of GI genotypes like GI.3[P13] to persist endemically and trigger epidemic surges highlights the critical need for sustained and enhanced surveillance of these strains.

Instead, it is shaped by a complex interplay of factors, including frequent recombination and host immune pressure, as suggested by Kobayashi et al. in their analysis of GI capsid evolution [39]. Collectively, these findings support the hypothesis that an accelerated evolutionary rate, partly driven by a proficient RdRp, enhances viral adaptability, potentially enabling escape from population immunity and thereby facilitating epidemic spread [40]. A highly active RdRp may increase the rate of mutation generation during replication, supplying the genetic variation—“mutational raw material”—for the VP1 gene. This variation can introduce novel amino acid changes in VP1 antigenic epitopes, altering the virus’s antigenic profile. Host immune pressure then acts on this variation, selecting and enriching mutations that confer an immune escape advantage. Thus, the replicative efficiency of the RdRp and the selective force of host immunity likely work in tandem to drive VP1 evolution, enabling the virus to continuously adapt within the host population [41].

Root-to-tip regression demonstrated a clear positive temporal signal in the GI.3 NoV RdRp gene (R^2^ = 0.5764), with consistent mutation accumulation patterns across the GI.3[P3], GI.3[P10], and GI.3[P13] genotypes, indicating a relatively stable and clock-like evolutionary pattern. In contrast, the VP1 gene exhibited markedly different evolutionary dynamics, with root-to-tip divergence distances mirroring the phylogenetic cluster structure observed in the ML tree. Further analysis revealed substantial intra-genotype variation: while GI.3[P3] VP1 (Cluster V) and GI.3[P13] VP1 (Cluster IV) showed positive mutation accumulation trends, GI.3[P13] VP1 (Cluster II) displayed a negative trend. The GI.3[P13] strains in Cluster II are predominantly concentrated in Asia, suggesting the establishment of a dominant regional transmission network, possibly amplified by person-to-person spread or foodborne outbreaks. This localized expansion may have created a population bottleneck, constraining VP1 genetic diversity [42]. At the same time, the VP1 protein carries major antigenic epitopes. While mutations in these regions can facilitate immune escape, excessive variation may reduce viral fitness. Thus, under relaxed immune pressure or to sustain transmission, VP1 may undergo reversion toward an ancestral, optimally adapted conformation [43], contributing to the observed negative evolutionary signal in this cluster. These cluster-specific evolutionary patterns suggest that VP1 evolution is influenced by complex selective pressures, including regional host immune responses and environmental factors, leading to divergent evolutionary trajectories among subclusters [44]. The more predictable evolutionary pattern of RdRp compared to the complex evolution of VP1 may provide a replicative advantage, though future studies with expanded datasets are needed to fully elucidate the intra-gene variability in both genomic regions.

Our biochemical data offer a direct functional explanation for these evolutionary observations. The significantly higher enzymatic activity and superior substrate affinity (lower K_m_) of the prevalent GI.3[P3] and GI.3[P13] RdRps provide a clear replicative advantage. This enhanced activity likely allows for more efficient viral RNA synthesis within the host, increasing viral load and potentially facilitating transmission. The observation that GI.3 RdRp requires higher manganese concentrations for optimal activity than those of GII RdRp [8,45], presenting a compelling biochemical hypothesis for the lower overall prevalence of GI NoVs. Given the low physiological Mn^2+^ concentration in the human body [46], the GII polymerase may be better adapted to achieve peak catalytic efficiency in the host environment.

The Michaelis constant (K_m_) represents the substrate concentration at which the enzymatic reaction velocity attains half of its maximum rate. A lower K_m_ value signifies higher affinity between the enzyme and the substrate, whereas a higher K_m_ value indicates reduced affinity. Accordingly, the kinetic parameters further refine this model: the high substrate affinity of GI.3[P13] RdRp suggests an advantage in environments with limited nucleotide pools, while the high V_max_ of GI.3[P3] RdRp indicates a superior capacity for rapid RNA production under ideal conditions. These distinct kinetic strategies may contribute to the co-circulation and success of both lineages.

This study has several limitations. First, as all sequences were sourced from GenBank, our study is subject to inherent public-database limitations—uneven spatiotemporal sampling, missing metadata, and variable sequence quality—that may introduce bias into the results. The analysis of the rare GI.3[P14] strain was constrained by scarce sequence availability. Nonetheless, investigating rare genotypes is valuable as they may be progenitors of future epidemic variants, helping to identify surveillance gaps. Second, norovirus research lacks mature, stable cell culture and animal models, restricting RdRp functional studies to in vitro systems that fail to recapitulate the complexity of in vivo viral replication, transmission, and host–virus interactions. Third, while we characterized RdRp enzymatic activity, its fidelity—closely linked to viral adaptability, pathogenicity—remains understudied, with unresolved structural bases underlying fidelity regulation.

Future work should address these gaps through three key avenues: (1) Expand the dataset and include a wider range of genotypes to further validate the reliability of our conclusions, fill surveillance gaps, and identify potential “reservoir genotypes” with epidemic potential. (2) Develop physiologically relevant model systems (e.g., organoid or humanized animal models) to systematically validate in vitro findings and unravel in vivo RdRp functions, and recapitulate complex host–virus interactions and immune responses. (3) Investigate RdRp fidelity and its structural determinants, integrating biophysical, structural, and functional assays to decipher how fidelity modulates viral evolution, adaptability, and pathogenicity. These efforts will deepen the understanding of norovirus pathogenicity, inform rational design of broad-spectrum antivirals and universal vaccines, and strengthen strategies for disease prevention and control.

## 5. Conclusions

In conclusion, this work establishes a clear correlation between RdRp enzymatic efficiency and GI.3 norovirus prevalence. Prevalent strains possess superior evolutionary rates, catalytic activity, and substrate affinity. We propose that enhanced RdRp function provides a replicative advantage and may accelerate the generation of VP1 diversity, upon which other selective forces act. This mechanistic link between polymerase activity and viral fitness offers a foundational model for understanding strain emergence and informs the development of antiviral strategies targeting viral replication.

## Figures and Tables

**Table 1:** Temporal distribution and genotype composition of GI.3 norovirus sequences till 2024.

	Year	<2000	2001–2005	2006–2010	2011–2015	2016–2020	2021–2025	Total
Genotype								
GI.3[P3]		8(21.6)	42(32.3)	7(10)	55(24.8)	111(34.4)	3(6.2)	226(27.2)
GI.3[P13]		2(5.4)	32(24.6)	5(7.1)	21(9.5)	96(29.5)	24(50)	180(21.1)
GI.3[P10]		2(5.4)	0	4(5.7)	3(1.3)	6(1.8)	3(6.2)	18(2.2)
GI.3[P14]		1(2.7)	0	0	3(1.3)	0	0	4(0.5)
GI.3[PNA]		0	2(1.5)	0	1(0.4)	0	0	3(0.4)
GI.3 *		24(64.8)	54(41.5)	54(77.1)	138(62.4)	112(34.4)	18(37.5)	400(48.1)
Total		37(100)	130(100)	70(100)	221(100)	325(100)	48(100)	831(100)

Data are *n* (%). GI.3 *: GI.3 NoV sequences without RdRp genotype information.

**Table 2:** Bayesian evolutionary rate estimates for GI.3 norovirus RdRp and VP1 genes.

Region	Nucleotide Evolutionary Rate (10-3 Substitutions/Site/Year)	*n* (%)
All RdRp gene	2.25 (1.75, 2.79)	83 (100)
All GI.3 VP1 gene	2.64 (1.97, 3.36)	154 (100)
GI.3[P3] RdRp gene	5.26 (4.03, 6.72)	38 (45.7)
GI.3[P10] RdRp gene	2.61 (1.93, 3.36)	5 (6.0)
GI.3[P13] RdRp gene	3.04 (1.83, 4.53)	38 (45.7)
GI.3[P14] RdRp gene	-	2 (2.4)
GI.3[P3] VP1 gene	3.76 (1.99, 5.59)	83 (53.9)
GI.3[P10] VP1 gene	0.12 (<0.01, 0.85)	5 (3.2)
GI.3[P13] VP1 gene	2.75 (2.41, 3.11)	63 (40.9)
GI.3[P14] VP1 gene	3.60 (3.19, 4.27)	3 (1.9)

The value in parentheses in the second column are 95% HPDs.

**Table 3:** Michaelis-Menten kinetic parameters of GI.3 norovirus RdRp variants.

Genotype	K_m_ (GTP), Michaelis-Menten Model(mM) at 30 °C	V_max_
GI.3[P3]	0.176	50,817
GI.3[P10]	0.198	32,704
GI.3[P13]	0.092	48,941
GI.3[P14]	0.273	30,541

“K_m_” refers to Michaelis constants; “V_max_” refers to maximum velocity.

## Data Availability

Data will be made available on request.

## Supplementary Materials

The following supporting information can be downloaded at https://www.mdpi.com/article/10.3390/microorganisms14010011/s1, Figure S1: SDS-PAGE analysis of four RdRp proteins from GI.3 NoV following induction; Figure S2: SDS-PAGE analysis of four RdRp proteins from GI.3 NoV following ion-exchange purification; Figure S3. Enzymatic characterization of GI.3 norovirus RdRp variants.; Table S1: GI.3 sequences collected from GenBank; Table S2: Optimal substitution models for nucleotides and amino acids for each genotype; Table S3: The marginal likelihoods estimated of molecular clock models and coalescent models; Table S4: Number of reported sequences of GI.3 stratified by region; Table S5: Comparison of evolutionary rates of GI.3[P3] and GI.3[P13] stratified by region and year. Supplementary File S1: GI.3 Reference Sequence.

## Abbreviations

The following abbreviations are used in this manuscript:

NoV	norovirus
RdRp	RNA-dependent RNA polymerase
VP1	major capsid protein
ORFs	open reading frames
HBGAs	histo-blood group antigens
NCBI	National Center for Biotechnology Information
ML	maximum likelihood
MCMC	Bayesian Markov Chain Monte Carlo
BIC	Bayesian information criterion
HPD	highest posterior density
ESS	effective sample size
bp	base pairs
nt	nucleotide
dsRNA	double-stranded RNA
RFU	relative fluorescence unit
K_m_	Michaelis constant
V_max_	maximum velocity
