# Supplementary material for: Superior RdRp Function Drives the Dominance of Prevalent GI.3 Norovirus Lineages
Source: Microorganisms. 2025 Dec 19;14(1):11. doi: 10.3390/microorganisms14010011 (PMC12844039; doi:10.3390/microorganisms14010011)
Supplement: Supplementary file 1 [file microorganisms-14-00011-s001.zip › Supplementary Materials/Supplementary Materials.pdf]

Supplementary Tables

Table S1: GI.3 sequences collected from GenBank.

Genotype	Sequences used for statistical description	Sequences used for RdRp evolutionary analysis	Sequences used for VP1 evolutionary analysis
GI.P3/GI.3	MH608286.1, MG557648.1, MG557650.1, KJ194510.1, PP594195.1, OP162337.1, LC122715.1, LC122716.1, LC122717.1, LC122718.1, LC122719.1, MW305489.1, MW305490.1, MW305494.1, MW305495.1, MW305500.1, MW305509.1, MW305510.1, MT089578.1, MT089579.1, MT089580.1, MT372463.1, MT372468.1, MT372470.1, MT031988.1, MN448477.1, NC_039897.1, MH218647.1, MH218659.1, MH218664.1, MH218722.1, MH218724.1, MH218725.1, MH218726.1, MH218727.1, MH218728.1, MH218729.1, KY934262.1, KJ196292.1, PQ657421.1,	MG557650.1_GI.P3-GI.3_Ethiopia_2016<br>MH608286.1_GI.P3-GI.3_Cameroon_2014<br>MT031988.1_GI.P3-GI.3_USA_2019<br>PP594195.1_GI.P3-GI.3_China_2024<br>MW305494.1_GI.P3-GI.3_Argentina_2004<br>MW305495.1_GI.P3-GI.3_Argentina_2005<br>KJ194510.1_GI.P3-GI.3_Netherlands_1995<br>MW305489.1_GI.P3-GI.3_USA_1988<br>MW305490.1_GI.P3-GI.3_USA_1988<br>MW305509.1_GI.P3-GI.3_USA_1988<br>MW305510.1_GI.P3-GI.3_USA_1988<br>KJ196292.1_GI.P3-GI.3_Japan_2007<br>NC_039897.1_GI.P3-GI.3_Japan_2007<br>LC122718.1_GI.P3-GI.3_Japan_2011<br>LC122719.1_GI.P3-GI.3_Japan_2012<br>MN448477.1_GI.P3-GI.3_USA_2013<br>MT372463.1_GI.P3-GI.3_USA_2016<br>MT372470.1_GI.P3-GI.3_USA_2015<br>MT372468.1_GI.P3-GI.3_USA_2015<br>LC122715.1_GI.P3-GI.3_Japan_2014	MW305494.1_GI.P3-GI.3_Argentina_2004<br>MT372463.1_GI.P3-GI.3_USA_2016<br>MT372470.1_GI.P3-GI.3_USA_2015<br>MT372468.1_GI.P3-GI.3_USA_2015<br>LC720155.1_GI.P3-GI.3_Japan_2014<br>LC122715.1_GI.P3-GI.3_Japan_2014<br>MN922658.1_GI.P3-GI.3_China_2016<br>MN922661.1_GI.P3-GI.3_China_2017<br>MN922648.1_GI.P3-GI.3_China_2016<br>MN922651.1_GI.P3-GI.3_China_2016<br>PP594195.1_GI.P3-GI.3_China_2024<br>MT031988.1_GI.P3-GI.3_USA_2019<br>MH608286.1_GI.P3-GI.3_Cameroon_2014<br>MG557650.1_GI.P3-GI.3_Ethiopia_2016<br>MW305509.1_GI.P3-GI.3_USA_1988<br>KJ194510.1_GI.P3-GI.3_Netherlands_1995<br>AF414403.1_GI.P3-GI.3_USA_1992<br>AF414405.1_GI.P3-GI.3_USA_1994<br>NC_039897.1_GI.P3-GI.3_Japan_2007<br>LC122718.1_GI.P3-GI.3_Japan_2011

	LC720153.1, LC720154.1,	LC122716.1_GI.P3-GI.3_Japan_2014	LC122719.1_GI.P3-GI.3_Japan_2012
	LC720155.1, LC720156.1,	LC122717.1_GI.P3-GI.3_Japan_2014	MN448477.1_GI.P3-GI.3_USA_2013
	MN922648.1, MN922649.1,	MT089578.1_GI.P3-GI.3_USA_2019	MT089578.1_GI.P3-GI.3_USA_2019
	MN922650.1, MN922651.1,	MT089579.1_GI.P3-GI.3_USA_2019	KY934262.1_GI.P3-GI.3_China_2015
	MN922652.1, MN922653.1,	MT089580.1_GI.P3-GI.3_USA_2019	LC378985.1_GI.P3-GI.3_Japan_2015
	MN922654.1, MN922655.1,	KY934262.1_GI.P3-GI.3_China_2015	OP162337.1_GI.P3-GI.3_Netherlands_2016
	MN922656.1, MN922657.1,	MW305500.1_GI.P3-GI.3_Argentina_2016	MH218664.1_GI.P3-GI.3_United_Kingdom_2015
	MN922658.1, MN922659.1,	MH218664.1_GI.P3-GI.3_United_Kingdom_2015	MH218722.1_GI.P3-GI.3_United_Kingdom_2014
	MN922660.1, MN922661.1,	OP162337.1_GI.P3-GI.3_Netherlands_2017	MH218729.1_GI.P3-GI.3_United_Kingdom_2015
	MN922662.1, MN922663.1,	MH218722.1_GI.P3-GI.3_United_Kingdom_2014	LC378984.1_GI.P3-GI.3_Japan_2015
	MN922664.1, MN922665.1,	MH218729.1_GI.P3-GI.3_United_Kingdom_2015	MH218647.1_GI.P3-GI.3_United_Kingdom_2015
	MN922666.1, MN922667.1,	MH218659.1_GI.P3-GI.3_United_Kingdom_2015	MH218659.1_GI.P3-GI.3_United_Kingdom_2015
	MN922668.1, MN922669.1,	MH218647.1_GI.P3-GI.3_United_Kingdom_2015	MH218727.1_GI.P3-GI.3_United_Kingdom_2015
	MN922670.1, MN922671.1,	MH218727.1_GI.P3-GI.3_United_Kingdom_2015	MH218724.1_GI.P3-GI.3_United_Kingdom_2014
	MN922672.1, MN922673.1,	MH218728.1_GI.P3-GI.3_United_Kingdom_2015	MH218726.1_GI.P3-GI.3_United_Kingdom_2014
	MN922674.1, MN922677.1,	MH218725.1_GI.P3-GI.3_United_Kingdom_2014	MH218725.1_GI.P3-GI.3_United_Kingdom_2014
	MN922678.1, MN922681.1,	MH218724.1_GI.P3-GI.3_United_Kingdom_2014	MH218728.1_GI.P3-GI.3_United_Kingdom_2015
	MN922686.1, MN922687.1,	MH218726.1_GI.P3-GI.3_United_Kingdom_2014	MW305500.1_GI.P3-GI.3_Argentina_2016
	MN922688.1, MN922689.1,		MN922650.1_GI.P3-GI.3_China_2016
	MN922690.1, MN922691.1,		MN922653.1_GI.P3-GI.3_China_2016
	MN922692.1, MN922695.1,		MN922649.1_GI.P3-GI.3_China_2015
	MN922696.1, MN922697.1,		MN922652.1_GI.P3-GI.3_China_2016
	MN922698.1, MN922699.1,		MN922654.1_GI.P3-GI.3_China_2016
	MN922700.1, MN922702.1,		LC378991.1_GI.P3-GI.3_Japan_2015
	MN922703.1, MN922704.1,		LC378989.1_GI.P3-GI.3_Japan_2015

	MN922706.1, MN922707.1, MN922708.1, MN922710.1, MN922711.1, MN922712.1, MN922715.1, MN922716.1, MN922717.1, MN922718.1, LC378984.1, LC378985.1, LC378986.1, LC378987.1, LC378988.1, LC378989.1, LC378990.1, LC378991.1, LC378992.1, LC378993.1, AF414403.1, AF414405.1, JX846929.1, PQ455501.1, PQ455504.1, PQ455507.1, PQ455509.1, PP733374.1, PP733375.1, PP733383.1, PP733385.1, OR780757.1, OR780758.1, OR780760.1, LC620998.1, LC621004.1, OM182811.1, OM182812.1, OM182813.1, OM182814.1, MW649128.1, MZ291636.1, MZ291637.1, MZ291638.1, MZ068114.1, MZ068116.1, MW800358.1, MW800359.1, MW800360.1, MW714855.1,		LC378990.1_GI.P3-GI.3_Japan_2015<br>LC378986.1_GI.P3-GI.3_Japan_2015<br>LC378988.1_GI.P3-GI.3_Japan_2015<br>LC720153.1_GI.P3-GI.3_Japan_2015<br>LC378992.1_GI.P3-GI.3_Japan_2015<br>LC720154.1_GI.P3-GI.3_Japan_2015<br>LC720156.1_GI.P3-GI.3_Japan_2015<br>MN922700.1_GI.P3-GI.3_China_2018<br>MN922678.1_GI.P3-GI.3_China_2018<br>MN922718.1_GI.P3-GI.3_China_2018<br>MN922662.1_GI.P3-GI.3_China_2017<br>MN922663.1_GI.P3-GI.3_China_2017<br>MN922698.1_GI.P3-GI.3_China_2018<br>MN922666.1_GI.P3-GI.3_China_2017<br>MN922677.1_GI.P3-GI.3_China_2018<br>MN922668.1_GI.P3-GI.3_China_2018<br>MN922691.1_GI.P3-GI.3_China_2018<br>MN922708.1_GI.P3-GI.3_China_2018<br>MN922659.1_GI.P3-GI.3_China_2017<br>MN922681.1_GI.P3-GI.3_China_2018<br>MN922686.1_GI.P3-GI.3_China_2018<br>MN922692.1_GI.P3-GI.3_China_2018<br>MN922706.1_GI.P3-GI.3_China_2018<br>MN922695.1_GI.P3-GI.3_China_2018<br>MN922707.1_GI.P3-GI.3_China_2018

	MW680989.1, MW680996.1, MW680997.1, MW658676.1, MW658677.1, MW658685.1, MW658686.1, MW647671.1, MW647676.1, MW647677.1, MW647678.1, MW647679.1, MW647680.1, MW644792.1, MW647649.1, MW578746.1, MW578753.1, MW578757.1, MW578759.1, MW578761.1, MW368602.1, MW368603.1, MW368604.1, MW368605.1, MW362458.1, MW362459.1, MW362460.1, MW325976.1, LC576687.1, MT492002.1, MT492062.1, MT492063.1, MT492064.1, MT492068.1, MT492069.1, MK387036.1, MK387047.1, MK387049.1, MK408526.1, MK408527.1, MK408528.1, MK280856.1, MK280857.1, MK280859.1, MK280861.1, MK280868.1, MK280872.1, MK280873.1, MK280874.1, MK280878.1,		MN922687.1_GI.P3-GI.3_China_2018<br>MN922699.1_GI.P3-GI.3_China_2018<br>MN922717.1_GI.P3-GI.3_China_2018<br>MN922712.1_GI.P3-GI.3_China_2018<br>MN922715.1_GI.P3-GI.3_China_2018<br>MN922697.1_GI.P3-GI.3_China_2018<br>MN922710.1_GI.P3-GI.3_China_2018<br>MN922669.1_GI.P3-GI.3_China_2018<br>MN922670.1_GI.P3-GI.3_China_2018<br>MN922711.1_GI.P3-GI.3_China_2018<br>MN922689.1_GI.P3-GI.3_China_2018<br>MN922702.1_GI.P3-GI.3_China_2018<br>MN922703.1_GI.P3-GI.3_China_2018

	MG585906.1, MG585911.1, MG585915.1, MG585917.1, MG585918.1, MG585919.1, MG585920.1, MG585921.1, MG585924.1, MG585931.1, MG585932.1, MG585933.1, MG585935.1, JF802508.1, JF802509.1, EF078281.1, KC119510.1, KC017784.1, EU085483.1, EU085497.1, EU085498.1, EU085499.1, EU085500.1, EU085501.1, EU085502.1, EU085503.1, EU085504.1, EU085505.1, EU085506.1, EU085507.1, EU085508.1, EU085509.1, EU085510.1, EU085511.1, EU085512.1, EU085513.1		
GI.P10/GI.3	OR648469.1, ON033826.1, MW305506.1, MW305507.1, MT008457.1, MZ291642.1, MZ068113.1, MW658674.1, MW658675.1, AB621909.1,	MW305507.1_GI.P10-GI.3_Saudi_Arabia_1990<br>OR648469.1_GI.P10-GI.3_USA_2023<br>MT008457.1_GI.P10-GI.3_China_2018<br>ON033826.1_GI.P10-GI.3_Spain_2018<br>MW305506.1_GI.P10-GI.3_Saudi_Arabia_1990	MW305507.1_GI.P10-GI.3_Saudi_Arabia_1990<br>OR648469.1_GI.P10-GI.3_USA_2023<br>MT008457.1_GI.P10-GI.3_China_2018<br>ON033826.1_GI.P10-GI.3_Spain_2018<br>MW305506.1_GI.P10-GI.3_Saudi_Arabia_1990

	KC970259.1, PQ455505.1, OR597885.1, PP389371.1, MW362457.1, MW255366.1, MT492061.1, KC970259.1		
GI.P13/GI.3	PQ582089.1, PP584624.1, OR084254.1, OR084255.1, JQ911594.1, PQ632216.1, PQ632217.1, LC848163.1, LC848164.1, LC848166.1, PP663093.1, PP564824.1, OR792774.1, OR792775.1, LC769686.1, LC769687.1, LC769690.1, LC769710.1, LC769711.1, OP162334.1, LC122714.1, MZ462929.1, MZ462930.1, MZ462931.1, MZ462932.1, MW305492.1, MW305493.1, MZ203488.1, MT008455.1, MT008456.1, MT526276.1, MT526277.1, MT451991.1, MT372466.1, MT372467.1, MK073887.1, MK073892.1, MK073893.1, MH218721.1, MH218723.1,	MW305492.1_GI.P13-GI.3_French_Guiana_1978<br>MW305493.1_GI.P13-GI.3_French_Guiana_1978<br>LC848163.1_GI.P13-GI.3_Thailand_2022<br>LC848164.1_GI.P13-GI.3_Thailand_2022<br>PP584624.1_GI.P13-GI.3_Thailand_2023<br>PQ582089.1_GI.P13-GI.3_China_2023<br>LC769686.1_GI.P13-GI.3_India_2019<br>LC769710.1_GI.P13-GI.3_India_2018<br>LC769711.1_GI.P13-GI.3_India_2018<br>LC769690.1_GI.P13-GI.3_India_2018<br>LC769687.1_GI.P13-GI.3_India_2019<br>PP663093.1_GI.P13-GI.3_USA_2023<br>PQ632216.1_GI.P13-GI.3_China_2019<br>PQ632217.1_GI.P13-GI.3_China_2019<br>LC122714.1_GI.P13-GI.3_Japan_2012<br>JQ911594.1_GI.P13-GI.3_Vietnam_2010<br>MZ203488.1_GI.P13-GI.3_USA_2012<br>MT008456.1_GI.P13-GI.3_China_2018<br>OR792774.1_GI.P13-GI.3_China_2017<br>MT372466.1_GI.P13-GI.3_USA_2015	LC769690.1_GI.P13-GI.3_India_2019<br>PP663093.1_GI.P13-GI.3_USA_2023<br>LC848163.1_GI.P13-GI.3_Thailand_2022<br>PP584624.1_GI.P13-GI.3_Thailand_2023<br>PQ582089.1_GI.P13-GI.3_China_2023<br>PP564824.1_GI.P13-GI.3_Thailand_2023<br>LC769686.1_GI.P13-GI.3_India_2019<br>LC769710.1_GI.P13-GI.3_India_2018<br>LC769711.1_GI.P13-GI.3_India_2018<br>LC769687.1_GI.P13-GI.3_India_2019<br>MN922741.1_GI.P13-GI.3_China_2019<br>PQ632216.1_GI.P13-GI.3_China_2019<br>MN922742.1_GI.P13-GI.3_China_2019<br>MZ203488.1_GI.P13-GI.3_USA_2012<br>JQ911594.1_GI.P13-GI.3_Vietnam_2010<br>MN922705.1_GI.P13-GI.3_China_2018<br>OR792774.1_GI.P13-GI.3_China_2017<br>MN922732.1_GI.P13-GI.3_China_2019<br>MT008456.1_GI.P13-GI.3_China_2018<br>MN922693.1_GI.P13-GI.3_China_2018

	MW445537.1, MN922675.1,	MT372467.1_GI.P13-GI.3_USA_2015	MN922679.1_GI.P13-GI.3_China_2018
	MN922676.1, MN922679.1,	MH218723.1_GI.P13-GI.3_United_Kingdom_2014	MN922675.1_GI.P13-GI.3_China_2018
	MN922680.1, MN922682.1,	MH218721.1_GI.P13-GI.3_United_Kingdom_2014	MN922680.1_GI.P13-GI.3_China_2018
	MN922683.1, MN922684.1,	MW445537.1_GI.P13-GI.3_China_2018	MN922726.1_GI.P13-GI.3_China_2019
	MN922685.1, MN922693.1,	OR792775.1_GI.P13-GI.3_China_2018	MN922737.1_GI.P13-GI.3_China_2019
	MN922694.1, MN922701.1,	MT008455.1_GI.P13-GI.3_China_2018	MN922729.1_GI.P13-GI.3_China_2019
	MN922705.1, MN922709.1,	OR084254.1_GI.P13-GI.3_China_2017	MN922734.1_GI.P13-GI.3_China_2019
	MN922713.1, MN922714.1,	OR084255.1_GI.P13-GI.3_China_2017	MN922736.1_GI.P13-GI.3_China_2019
	MN922719.1, MN922720.1,	MT451991.1_GI.P13-GI.3_USA_2014	MN922725.1_GI.P13-GI.3_China_2019
	MN922721.1, MN922722.1,	MT526276.1_GI.P13-GI.3_USA_2014	MN922728.1_GI.P13-GI.3_China_2019
	MN922723.1, MN922724.1,	MT526277.1_GI.P13-GI.3_USA_2014	MN922733.1_GI.P13-GI.3_China_2019
	MN922725.1, MN922726.1,	MZ462929.1_GI.P13-GI.3_Mexico_2016	MN922684.1_GI.P13-GI.3_China_2018
	MN922727.1, MN922728.1,	MZ462930.1_GI.P13-GI.3_Mexico_2016	MN922720.1_GI.P13-GI.3_China_2019
	MN922729.1, MN922730.1,	MZ462932.1_GI.P13-GI.3_Mexico_2017	MN922727.1_GI.P13-GI.3_China_2019
	MN922731.1, MN922732.1,	MK073887.1_GI.P13-GI.3_USA_2016	MN922722.1_GI.P13-GI.3_China_2019
	MN922733.1, MN922734.1,	OP162334.1_GI.P13-GI.3_Netherlands_2017	MN922740.1_GI.P13-GI.3_China_2019
	MN922735.1, MN922736.1,	MK073892.1_GI.P13-GI.3_USA_2016	LC122714.1_GI.P13-GI.3_Japan_2012
	MN922737.1, MN922738.1,	MK073893.1_GI.P13-GI.3_USA_2016	JN603244.1_GI.P13-GI.3_Sweden_2008
	MN922739.1, MN922740.1,		JN183164.1_GI.P13-GI.3_Sweden_2008
	MN922741.1, MN922742.1,		MW305492.1_GI.P13-GI.3_French_Guiana_1978
	MN428922.1, JN183164.1,		MT372467.1_GI.P13-GI.3_USA_2015
	JN603244.1, PQ455502.1,		MT372466.1_GI.P13-GI.3_USA_2015
	PQ455513.1, PQ455516.1,		MH218723.1_GI.P13-GI.3_United_Kingdom_2014
	PQ455517.1, OR523294.1,		MT526276.1_GI.P13-GI.3_USA_2014
	OR523295.1, OR597874.1,		MH218721.1_GI.P13-GI.3_United_Kingdom_2014

	OR597877.1, OR597888.1, PQ097766.1, PP733364.1, PP733367.1, PP733378.1, PP733379.1, PP733381.1, PP391263.1, PP391264.1, OR780022.1, OR780023.1, OR780024.1, OR780025.1, OR681033.1, OR681034.1, OR681035.1, OR252328.1, OR088506.1, OP156603.1, OP156604.1, OQ928377.1, OQ928378.1, OQ880479.1, MW686747.1, OM182815.1, OK147888.1, OK147889.1, MZ470610.1, MZ470611.1, MZ268167.1, MZ268168.1, MZ220453.1, MW714856.1, MW686941.1, MW686942.1, MW686943.1, MW686944.1, MW686945.1, MW680977.1, MW680979.1, MW680981.1, MW680987.1, MW680988.1, MW680990.1, MW680998.1, MW680999.1, MW681000.1, MW681001.1, MW658680.1,		MZ462929.1_GI.P13-GI.3_Mexico_2016<br>MZ462930.1_GI.P13-GI.3_Mexico_2016<br>OP162334.1_GI.P13-GI.3_Netherlands_2016<br>MK073887.1_GI.P13-GI.3_USA_2016<br>MK073892.1_GI.P13-GI.3_USA_2016<br>MW445537.1_GI.P13-GI.3_China_2018<br>MT008455.1_GI.P13-GI.3_China_2018<br>MN922682.1_GI.P13-GI.3_China_2018<br>MN922683.1_GI.P13-GI.3_China_2018<br>MN922713.1_GI.P13-GI.3_China_2018<br>MN922709.1_GI.P13-GI.3_China_2018<br>MN922714.1_GI.P13-GI.3_China_2018<br>OR084254.1_GI.P13-GI.3_China_2017<br>OR084255.1_GI.P13-GI.3_China_2017<br>OR792775.1_GI.P13-GI.3_China_2018<br>MN922721.1_GI.P13-GI.3_China_2019<br>MN922738.1_GI.P13-GI.3_China_2019

	MW658681.1, MW658687.1, MW658690.1, MW647667.1, MW644785.1, MW644790.1, MW578736.1, MW578738.1, MW578741.1, MW578743.1, MW578744.1, MW578747.1, MW578762.1, MW578763.1, MW578764.1, MW492586.1, MW397135.1, MW362456.1, MW356810.1, MW326131.1, MW326134.1, MW325973.1, MW255382.1, LC577862.1, MT491999.1, MT492003.1, MT492067.1, MN318341.1, MN156316.1, MK692747.1, MH393582.1, MK280865.1, MK280870.1, MK280880.1, MH828423.1, MH393673.1, MH393675.1, KP963773.1, LC147090.1, JN603247.1		
GI.P14/GI.3	AB187514.1, MH218730.1, KM289169.1, MN818798.1	AB187514.1_GI.P14-GI.3_Japan_1979<br>MH218730.1_GI.P14-GI.3_United_Kingdom_2015	KM289169.1_GI.P14-GI.3_Germany_2014<br>MH218730.1_GI.P14-GI.3_United_Kingdom_2015<br>AB187514.1_GI.P14-GI.3_Japan_1979
GI.PNA/GI.3	MW800617.1,	-	-

	MW578758.1, MW578760.1		
GI.3*	MK511873.1, MK511874.1, LC209683.1, LC209684.1, LC209685.1, LC048886.1, LC048958.1, AB985479.1, AB985485.1, KC911692.1, LC312748.1, LC312754.1, LC150707.1, AB684666.1, AB684673.1, AB684674.1, AB684729.1, KU973910.1, KU973913.1, KC820487.1, KC752591.1, JN699047.1, JQ943958.1, JQ943965.1, JQ943966.1, JQ943967.1, JQ943968.1, JQ943969.1, JF921986.1, JF921987.1, AB545078.1, AB545084.1, AB545101.1, AB545129.1, AB545137.1, AB545139.1, AB545172.1, AB545178.1, AB545192.1, AB545255.1, AB545279.1, AB545280.1, AB545282.1, AB545288.1, AB545291.1, AB545300.1,	-	-

	AB545341.1, AB545346.1, AB545356.1, AB545357.1, AB545385.1, EF363699.1, EF630429.1, EF630430.1, EF630431.1, DQ004614.1, DQ004615.1, DQ004617.1, DQ004616.1, DQ004618.1, DQ004619.1, DQ004620.1, DQ004621.1, DQ004622.1, DQ004623.1, DQ004624.1, DQ004625.1, DQ004626.1, DQ004627.1, DQ004628.1, DQ004629.1, DQ004630.1, DQ004632.1, DQ004631.1, DQ004634.1, DQ004633.1, DQ004635.1, DQ004636.1, DQ004637.1, DQ004655.1, AY237482.1, AY237483.1, PV022783.1, PV022784.1, PV022785.1, PV022788.1, PV022789.1, PV022790.1, LC847999.1, LC848000.1, LC848019.1, LC848032.1, PP753793.1, PP753795.1, PP753798.1, PP753804.1,		

	PP753807.1, PP753808.1, PP753812.1, PP753813.1, PP753816.1, PP753817.1, PP753821.1, OR367326.1, OQ919251.1, OQ919253.1, OQ919255.1, OQ919256.1, LC764809.1, OP862432.1, OP862363.1, OP862365.1, ON951688.1, ON681575.1, ON681576.1, ON681577.1, ON681578.1, ON681579.1, ON681580.1, ON681581.1, ON681582.1, ON681583.1, ON681584.1, ON681585.1, ON681586.1, ON197358.1, ON197359.1, ON197360.1, MW385950.1, MW385951.1, MW385953.1, OM281062.1, MW642197.1, MZ735697.1, OK493614.1, LC544078.1, MZ702939.1, MZ702940.1, MZ702941.1, MZ702951.1, MZ702952.1, MZ702953.1, MZ702954.1, MZ702955.1,		

	MZ702956.1, MZ702957.1, MZ702958.1, MZ702959.1, MZ702960.1, MZ702961.1, MZ702962.1, MZ702963.1, MZ702964.1, MZ702966.1, MZ702967.1, MZ702970.1, MZ702971.1, MZ702972.1, MW444889.1, MT322850.1, MT322852.1, MT322855.1, MT322856.1, MW513431.1, MW513435.1, LC529933.1, LC573321.1, LC573323.1, LC573330.1, LC573333.1, LC573338.1, LC573394.1, LC573399.1, LC573401.1, LC573402.1, LC521519.1, LC521521.1, LC521523.1, LC521524.1, LC521547.1, LC521581.1, LC521589.1, LC521601.1, MK589368.1, MK589369.1, MK589370.1, MK589371.1, MK589372.1, MK589373.1, MK589374.1, MK589375.1, MK589376.1, MK589377.1, MK589379.1,		

	MK589380.1, MK589381.1, MN263857.1, MN263858.1, MN263859.1, MN263860.1, MN263861.1, MK246664.1, MK681452.1, MK382381.1, MK101200.1, MK101201.1, MK101202.1, MK101203.1, MH744735.1, MF818329.1, KY040050.1, KY040051.1, KY040052.1, KX816577.1, KY611587.1, MF166784.1, LC160140.1, LC160141.1, LC160142.1, LC160144.1, LC160145.1, LC160146.1, KX255562.1, KX255563.1, KX255564.1, KX255565.1, KX255566.1, KX255567.1, KX255568.1, KX255569.1, KX255570.1, KX255571.1, KX255572.1, KX255573.1, KX255574.1, KX255575.1, KX255576.1, KX255577.1, KX255578.1, KX255579.1, KX255580.1, KX255581.1, KX255582.1, KX255583.1,		

	KU291235.1, KU291236.1, KU291237.1, KX017985.1, KX608874.1, KU950315.1, KU950319.1, KT382965.1, KT382967.1, KT382968.1, KT382969.1, LC169534.1, LC169535.1, LC169536.1, LC169537.1, KX130603.1, KX154755.1, KT239552.1, KT239556.1, KT239557.1, LC089053.1, LN612532.1, LN612533.1, KT150978.1, KT150979.1, KT150986.1, KT150990.1, KT150994.1, KT150995.1, KT150997.1, KT151001.1, KT151002.1, KT151004.1, KT151006.1, KT151007.1, KT151009.1, KT151010.1, KR904255.1, KR904258.1, KR904259.1, KR904260.1, KR904261.1, KR904262.1, KR904265.1, KR904266.1, KR904270.1, KR904273.1, KR904274.1, KR904275.1, KR904277.1,		

	KR904278.1, KR904279.1, KR904281.1, KP762417.1, KP762419.1, KP753279.1, KP753280.1, LC032469.1, KP064096.1, KM349492.1, KM349494.1, KM017952.1, KM017953.1, KM017954.1, KJ938997.1, KF569765.1, KF569769.1, KF569770.1, KF569771.1, KF569773.1, KF569774.1, KF569777.1, KF569780.1, KF569781.1, KF569782.1, KF569783.1, KF569794.1, KF475954.1, KF475955.1, KF475956.1, KF475957.1, KF361441.1, KF361442.1, KJ152204.1, KF279374.1, KF279377.1, JX416389.1, KC970247.1, KC783710.1, KC783714.1, KC783715.1, KC783717.1, KC783719.1, KC783720.1, KC783722.1, KC413368.1, KC413369.1, KC413370.1, KC413388.1, KC413389.1,		

	KC413390.1, KC413391.1,<br>KC413392.1, KC413393.1,<br>KC413394.1, JN699048.1,<br>JN699049.1, JN699050.1,<br>JX898883.1, JX683012.1,<br>JX683013.1, JQ743330.1,<br>FJ711163.1, FJ711164.1,<br>FJ711165.1, GU132469.1,<br>GU132470.1, GQ487572.1,<br>OR666410.1, OR666411.1,<br>OR674242.1, OR674243.1,<br>PP851054.1, OR780759.1,<br>OM372602.1, OM372603.1,<br>OM372604.1, OM372605.1,<br>OM372606.1, OM372607.1,<br>OM372609.1, OM372611.1,<br>OM372612.1, OM372614.1,<br>OM372615.1, MZ227012.1,<br>MW659097.1, MT928712.1,<br>MT860989.1, MW647668.1,<br>MN818799.1, MK280879.1,<br>MH107008.1, KY427653.1,<br>HE716741.1, HE716743.1,<br>HE716744.1, HE716745.1,<br>HE716746.1, HE716747.1,		

	HE716748.1, AB685382.1, GU138161.3, MW305491.1, MW558948.1, KT781411.1		

GI.3*: GI.3 NoV sequences without RdRp genotype information.

Table S2: Optimal substitution models for nucleotides and amino acids for each genotype.

Genotype	Optimal substitution models for nucleotides	Optimal substitution models for amino acids
All RdRp gene	GTR+G+I	JTT+G+I
P3-type RdRp	GTR+G	-
P10-type RdRp	TN93+G	-
P13-type RdRp	TN93+G	-
All GI.3 VP1 gene	GTR+G+I	JTT+G+I
GI.P3/GI.3 VP1	TN93+G+I	-
GI.P10/GI.3 VP1	TN93+G+I	-
GI.P13/GI.3 VP1	GTR+G+I	-
GI.P14/GI.3 VP1	TN93	-

Table S3: The marginal likelihoods estimated of molecular clock models and coalescent models.

Model of rate variation	Coalescent tree prior	Log marginal likelihood								
		All RdRp gene	P3-type RdRp	P10-type RdRp	P13-type RdRp	All GI.3 VP1 gene	GI.P3/GI.3 VP1	GI.P10/GI.3 VP1	GI.P13/GI.3 VP1	GI.P14/GI.3 VP1
Strict clock	Constant size	-13557.99	-6984.06	**-3609.69**	-5601.30	-21037.06	-10798.98	**-4858.55**	-9790.49	-3086.68
Strict clock	Exponential growth	-13554.25	-6984.34	-3610.31	-5606.24	-21010.96	-10792.39	-4871.05	-9768.67	-
Strict clock	Bayesian skyline	-13538.89	-6983.88	-3613.57	-5601.07	-20979.93	-10777.17	-4858.73	**-9750.18**	-3086.82
Uncorrelated relaxed clock	Constant size	-13551.51	-6981.33	-3615.07	-5600.28	-20954.33	-10753.86	-4865.75	-9765.52	**-3085.45**
Uncorrelated relaxed clock	Exponential growth	-13552.87	-6981.19	-3614.82	-5604.73	-20943.36	-10756.09	-4868.86	-9774.36	-
Uncorrelated relaxed clock	Bayesian skyline	**-13537.04**	**-6977.26**	-3615.03	**-5598.11**	**-20919.69**	**-10750.19**	-4868.09	-9754.06	-3088.06

The maximum log marginal likelihood values for each dataset, obtained via path sampling, are displayed in bold. A larger log marginal likelihood value indicates a superior model fit. For the present analysis, each dataset was analyzed using its best-fitting model.

Table S4：Number of reported sequences of GI.3 stratified by region.

Region<br>Genotype	Asia	Europe	Oceania	North America	South America	Africa	**Total**
GI.P3/GI.3	124(25.5)	42(34.7)	26(40.0)	16(29.1)	13(25.5)	5(9.4)	226(27.2)
GI.P13/GI.3	130(26.7)	15(12.4)	8(12.3)	14(25.5)	12(23.5)	1(1.9)	180(21.1)
GI.P10/GI.3	14(2.9)	2(1.7)	0	1(1.8)	1(2.0)	0	18(2.2)
GI.P14/GI.3	1(0.2)	2(1.7)	0	1(1.8)	0	0	4(0.5)
GI.PNA/GI.3	3(0.6)	0	0	0	0	0	3(0.4)
GI.3*	214(44.0)	60(49.6)	31(47.7)	23(41.8)	25(49.0)	47(88.7)	400(48.1)
**Total**	486(100)	121 (100)	65(100)	55 (100)	51 (100)	53(100)	831(100)

Data are n(%). GI.3*: GI.3 NoV sequences without RdRp genotype information.

Table S5: Comparison of evolutionary rates of GI.3[P3] and GI.3[P13] stratified by region and year.

Region	Nucleotide evolutionary rate (10-3 substitutions/site/year)		Year	Nucleotide evolutionary rate (10-3 substitutions/site/year)	
	GI.3[P3]	GI.3[P13]		GI.3[P3]	GI.3[P13]
Aisa	4.33 (1.38, 8.36)	2.30 (1.47, 3.07)	2011-2015	8.11 (0.71, 19.90)	0.48 (<0.01, 3.29)
Europe	3.46 (0.03, 6.60)	0.81 (<0.01, 6.10)	2016-2020	2.50 (0.96, 4.52)	2.71 (1.37, 4.33)

Values in parentheses are 95% HPDs.

Supplementary Figures

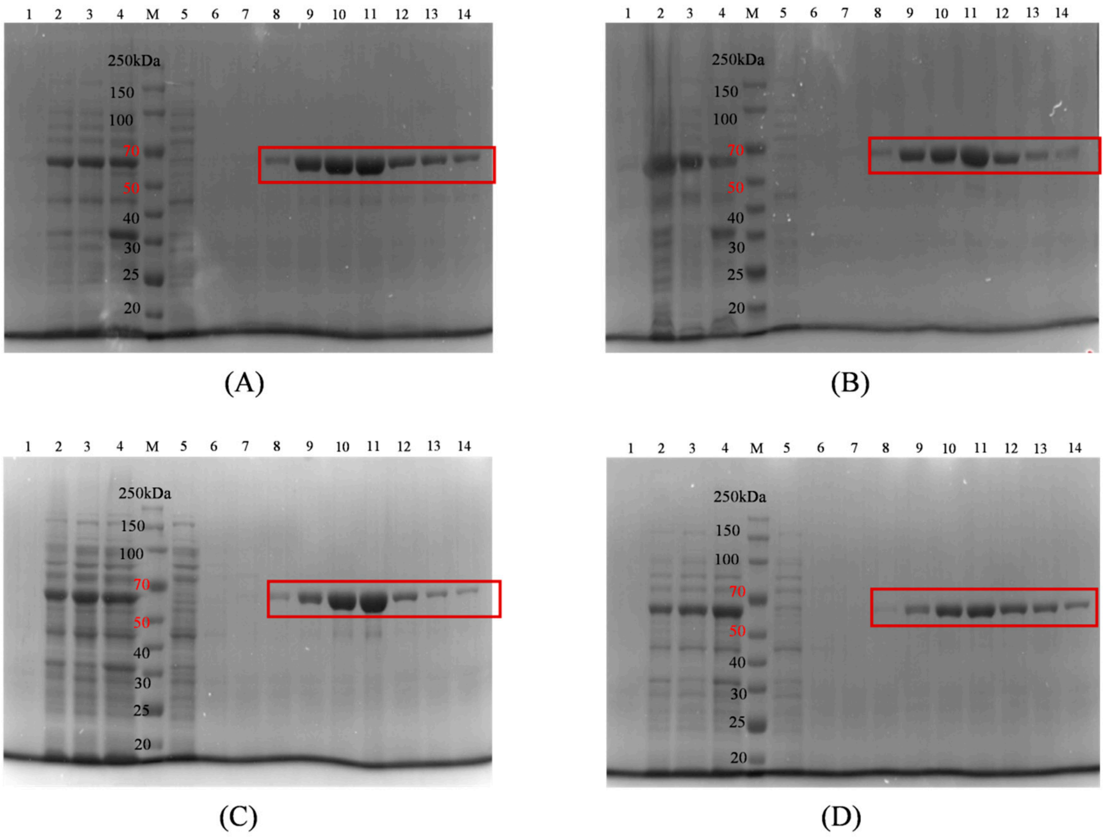

Figure S1. SDS-PAGE analysis of four RdRp proteins from GI.3 NoV following induction. (A–D) M, protein marker; Lane 1, total bacterial lysate prior to induction; Lane 2, total bacterial lysate post-induction; Lane 3, supernatant following sonication; Lane 4, pellet following sonication; Lane 5, flow-through from Ni-NTA column; Lanes 6–14, imidazole gradient elution fractions. (A) GI.P3; (B) GI.P10; (C) GI.P13; (D) GI.P14.

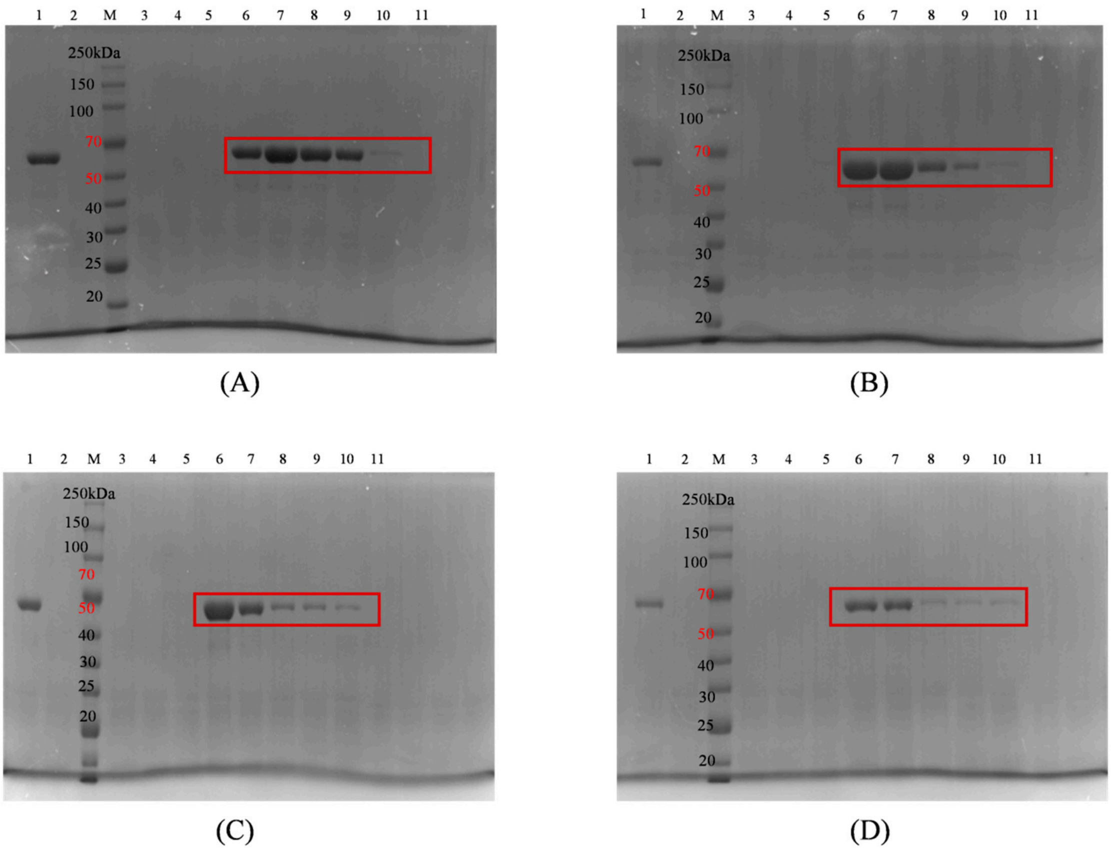

Figure S2. SDS-PAGE analysis of four RdRp proteins from GI.3 NoV following ion-exchange purification. (A–D) M, protein marker; Lane 1, protein solution after ultrafiltration concentration; Lane 2, flow-through following ion-exchange chromatography; Lanes 3–4, wash with 0 M NaCl; Lanes 5–14, elution with 0.25–1 M NaCl. (A) GI.P3; (B) GI.P10; (C) GI.P13; (D) GI.P14.

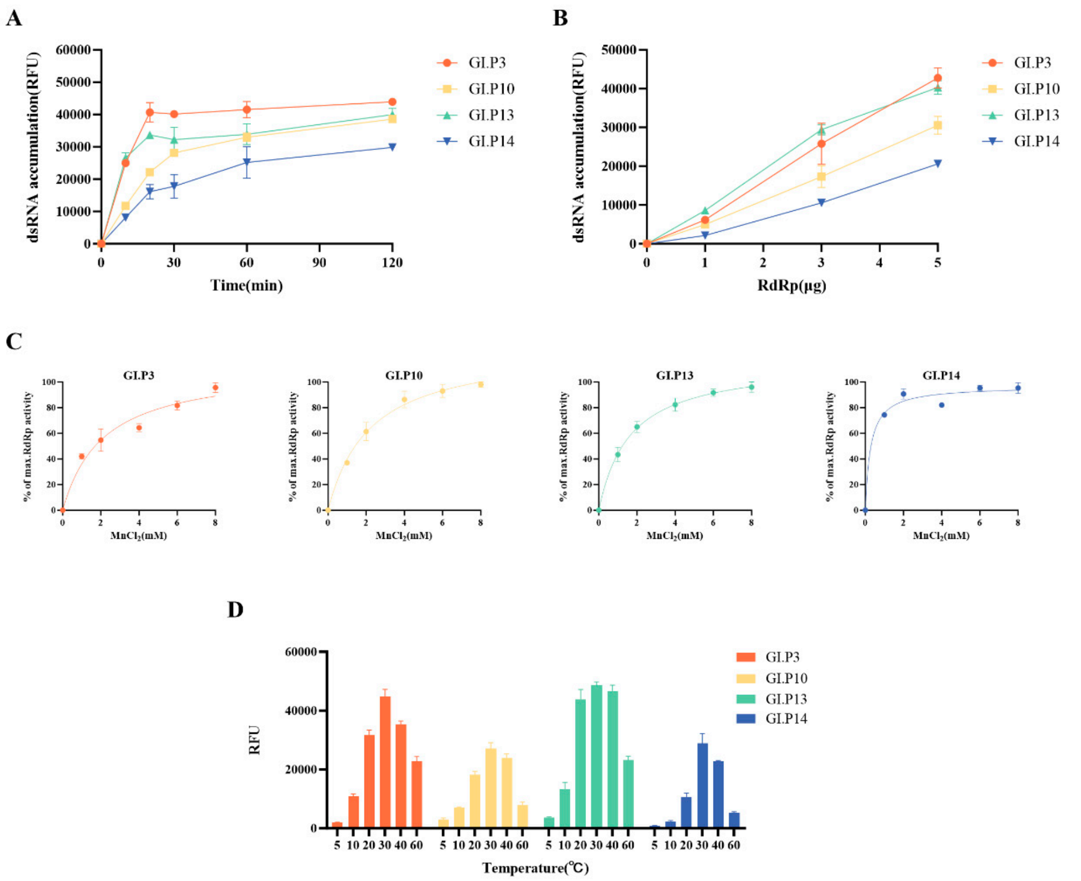

Figure S3. Enzymatic characterization of GI.3 norovirus RdRp variants. dsRNA synthesis was measured under varying (A) reaction time, (B) enzyme concentration, (C) Mn^{2+} concentration, (D) temperature for genotype comparison.

Supplementary Materials

A nucleotide sequence of norovirus GI.3 is provided as a reference, stored in a FASTA-format file (named "GI.3 Reference Sequence"). The full length of the sequence comprises 7,699 nucleotides, with positions 3,881–5,410 corresponding to the RdRp region and positions 5,411–7,031 to the VP1 region.
